# Yishen paidu pills attenuates 5/6 nephrectomy induced kidney disease via inhibiting the PI3K/AKT/mTOR signaling pathway

**DOI:** 10.3389/fphar.2024.1510098

**Published:** 2024-11-28

**Authors:** Saiji Liu, Yiling Cao, Qian Yuan, Yaru Xie, Yuting Zhu, Lijun Yao, Chun Zhang

**Affiliations:** Department of Nephrology, Union Hospital, Tongji Medical College, Huazhong University of Science and Technology, Wuhan, China

**Keywords:** network pharmacology, transcriptomics, 5/6 nephrectomy, PI3K/AKT/mTOR, yishen paidu pills

## Abstract

**Introduction:**

Chronic kidney disease (CKD) is a substantial global health issue with high morbidity and mortality. Yishen Paidu Pills (YSPDP) are effective concentrated water pills composed of four herbs developed by Wuhan Union Hospital to treat CKD. However, the mechanism of YSPDP action is largely unknown. This study combined metabolomics, network pharmacology, transcriptomics, and experimental verification to elucidate and identify the effects and potential mechanisms of YSPDP against CKD.

**Methods:**

Firstly, we used metabolomics analyses to identify the chemical components of YSPDP. Then, network pharmacology was conducted and indicated the predicted signaling pathways regulated by YSPDP. Next, we conducted a 5/6 subtotal nephrectomy (5/6 SNx) rat model and treated these rats with YSPDP or Losartan for 10 weeks to evaluate the effect of YSPDP on CKD. To further analyze the underlying mechanism of YSPDP in CKD, the kidney tissues of 5/6 SNx rats treated with vehicle and YSPDP were performed with transcriptome sequencing. Finally, the western blot was performed to validate the signaling pathways of YSPDP against CKD.

**Results:**

Twenty-four classes of chemicals were identified by metabolomics in YSPDP. YSPDP markedly hindered CKD progression, characterized by the restoration of body weight and serum albumin levels, improved renal function, diminished tissue injury, and hampered renal fibrosis in 5/6 SNx rats. The efficacy of YSPDP in ameliorating the progression of CKD was comparable to that of losartan. Furthermore, network pharmacology, transcriptomics, and functional enrichment analysis indicated the PI3K/AKT/mTOR signaling pathway was the key pathway regulated by YSPDP. Western blot validated the inhibition of PI3K/AKT/mTOR signaling in the kidney of 5/6 SNx rats treated by YSPDP.

**Conclusion:**

The study identified the chemicals of YSPDP and revealed that YSPDP prevented the progression of CKD by inhibiting PI3K/AKT/mTOR signaling in 5/6 SNx rats.

## 1 Introduction

Chronic kidney disease (CKD) is a substantial global health issue with high morbidity and mortality. The worldwide prevalence of CKD ranges from 8% to 16% (Jha et al., 2013), with CKD considered to become the fifth most prevalent chronic condition by 2040 (Foreman et al., 2018). Although diabetes mellitus, hypertension, and glomerulonephritis are recognized as the major causes of CKD, multiple other risk factors, including dyslipidemia, ischemia, obesity, infection, toxins, as well as autoimmune and inflammatory diseases, also contribute to the development and progression of CKD (Snively and Gutierrez, 2004; Xie et al., 2018). The pathological process of CKD is characterized by progressive loss of renal function and extensive renal fibrosis caused by massive deposition of extracellular matrix (ECM), which mainly includes glomerulosclerosis and renal interstitial fibrosis, eventually leading to end-stage renal disease (ESRD) (Angela et al., 2017; Zhao, 2019). The treatment options for CKD in the international medical community are limited. Angiotensin II receptor blockers or angiotensin-converting enzyme inhibitors are typically employed as first-line therapies to effectively delay the progression of CKD (Lambers Heerspink and de Zeeuw, 2013; Ruiz-Ortega et al., 2020). However, some patients are resistant to renin-angiotensin system inhibitors or hardly tolerate severe side effects from these drugs. Thus, it’s critical to find novel drugs that are effective in preventing and treating CKD. The efficacy of traditional Chinese medicine (TCM), an integral component of complementary and alternative medicine, in safeguarding human health has been demonstrated over millennia. Both preclinical investigations and clinical trials have illustrated the potential of TCM therapy in managing CKD, specifically in ameliorating proteinuria, mitigating the adverse effects, and reducing the risk of ESRD by 60% (Wojcikowski et al., 2006; Lin et al., 2015). Yishen Paidu Pills (YSPDP) are concentrated water pills developed by Wuhan Union Hospital based on folk prescriptions and modern medical theories, This pill is composed of four Chinese herbals which include *Rheum officinale, Astragalus membranaceus, Bombyx batryticatus,* and *Hirudo* (Deng et al., 2019), which collectively contribute to the nourishing of Qi and kidney, ascending lucidity and descending turbidity, activating blood circulation and removing blood stasis. YSPDP has been used in our hospital to treat CKD for 20 years, and clinical application has proven efficacy in protecting renal function (Jie et al., 2012; Deng et al., 2019). Despite its widespread use in CKD treatment, the underlying mechanism of its action remains unclear.

Network pharmacology (NP) is a novel field that blends biology, pharmacology, and informatics (Hopkins, 2007), which has recently emerged as a scientific approach for investigating the relationship between diseases and components (Nogales et al., 2022; Zhao et al., 2023). Transcriptomics, a method utilized to examine an organism’s transcriptome, which encompasses all its RNA transcripts, has been instrumental in identifying differentially expressed genes (DEGs) in individuals (Finotello and Di Camillo, 2015). Thus, the integration of network pharmacology and transcriptomics holds promise for effectively discerning the active constituents and molecular mechanisms underlying the therapeutic efficacy of TCM in treating diseases.

In this study, we evaluated the effect of YSPDP on CKD by constructing an animal model of 5/6 subtotal nephrectomy (5/6 SNx) revealing the molecular mechanism of YSPDP in treating CKD at the level of “ingredients-core target-pathway” by integrating NP and transcriptomics, and finally verified the potential molecular mechanism using experiments.

## 2 Materials and methods

### 2.1 Drugs and reagents

YSPDP was purchased from Wuhan Union Hospital (Hubei Medicine Number Z20181033, production batch number: 20220504). Losartan potassium tablet, a commonly used renin-angiotensin system inhibitor for CKD, was chosen as the positive control and obtained from Merck Sharp & Dohme Limited (Hangzhou, China). Both YSPDP and Losartan were ground into powder and mixed with ddH2O sufficiently before gavage.

### 2.2 Metabolomics profiling

Metabolomics profiling was supported by Metware Biotechnology Co., Ltd. (Wuhan, China). Liquid nitrogen was used to freeze-dry and crush the YSPDP. Afterward, 20 mg of the lyophilized powder was reconstituted in A 400 μL solution (Methanol: Water = 7:3, V/V). After shaking and centrifugation, the supernatant was filtered for analysis on the UPLC-MS/MS system. A UPLC system (ExionLC AD) coupled with a quadrupole-time-of-flight mass spectrometer (TripleTOF 6,600, AB SCIEX) was used to characterize chemical components in YSPDP. Chromatographic and mass spectrometry acquisition conditions and related data acquisition instrument systems are shown in Supplementary File S1.

### 2.3 Network pharmacology

#### 2.3.1 Screening of active compounds and targets of YSPDP

According to the drug description of YSPDP, it consists of *Rheum officinale, Astragalus membranaceus, Bombyx batryticatus,* and *Hirudo*. The active compounds and corresponding targets of *Rheum officinale* and *Astragalus membranaceus* were screened using the Traditional Chinese Medicine Systems Pharmacology database (TCMSP, https://tcmspw.com/tcmsp.php) and The Encyclopedia of Traditional Chinese Medicine database (ETCM, http://www.tcmip.cn/ETCM/index.php/Home/Index/) (Ru et al., 2014; Xu et al., 2019). *Bombyx batryticatus* and *Hirudo* are animal-based traditional Chinese medicines, so their chemical components and targets are collected through a literature search. Effective compounds were chosen based on a criterion requiring an oral bioavailability (OB) according to Veber’s filter, a drug-likeness (DL) following Lipinski rule of 5, and a high gastrointestinal (GI) absorption (Lipinski et al., 2001; Veber et al., 2002; Daina and Zoete, 2016). Then we imported the chemical components obtained into the Swiss Target Prediction database (https://www.expasy.org/resources/swisstargetprediction) to search for potential targets of YSPDP (Duvaud et al., 2021). When constructing the component-target network, molecules that were not predicted as targets by the Swiss Target Prediction database were not displayed, and chemical components contained in multiple monomers were only displayed in one monomer.

#### 2.3.2 Collection of CKD-related genes

The information on CKD-related genes was collected from the following resources. The Online Mendelian Inheritance in Man database (OMIM, https://www.nslij-genetics.org/omim/), DisGeNET database (http://www.disgenet.org/), National Center for Biotechnology Information database (NCBI, https://www.ncbi.nlm.nih.gov/), and Genecards database (https://www.genecards.org/) were adopted by search “CKD” and extracting the corresponding gene sets (Hamosh et al., 2005; Barrett et al., 2012; Stelzer et al., 2016; Piñero et al., 2020). Venn diagrams were then mapped to identify the intersection of CKD-related potential targets with drug component-related targets to serve as core targets for subsequent YSPDP treatment of CKD applied analysis.

#### 2.3.3 GO and KEGG analysis

The selected core targets were imported into The Database for Annotation, Visualization and Integrated Discovery (DAVID, https://david.ncifcrf.gov/) for GO enrichment analysis and KEGG pathway analysis (Jiao et al., 2012).

#### 2.3.4 Protein-protein interaction (PPI) network construction

The core targets were imported to the STRING database 11.5 (https://cn.string-db.org/) to construct the PPI network with the scoring condition to > 0.7 (Szklarczyk et al., 2021). The results obtained from STRING were uploaded into Cytoscape 3.9.0 software to visualize.

#### 2.3.5 Construction of the “component-targets-pathway” network

The components, core targets, and KEGG pathway data were used to construct a visualized “component-core targets-pathway” (C-T-P) regulatory network using Cytoscape 3.9.0 software.

### 2.4 Experimental protocols for animals

Animal studies were conducted following the National Institutes of Health (NIH) Guidelines for the Use and Care of Laboratory Animals and approved by Ethics Committee of Huazhong University of Science and Technology. 50 eight-week-old male Sprague-Dawley rats were purchased from Charles River (Beijing, China). The rats were kept in controlled conditions, with a temperature maintained at 23°C ± 2°C and humidity ranging from 30% to 70%, following a 12-hour dark/light cycle. They were provided *ad libitum* access to standard mouse chow and tap water. One week after purchase, animals were randomly assigned to 5 groups with 10 in each group: Sham; 5/6 SNx + Vehicle; 5/6 SNx + Losartan; 5/6 SNx + YSPDP-L; 5/6 SNx + YSPSP-H. The 5/6 SNx operation was performed as follows: amputation of the poles of the left kidney at week 1, followed at week 2 by uninephrectomy (Uni-Nx) of the remaining kidney. Sham operations were performed at the same time points. At 2 weeks after surgery, Rats in the Losartan group were gavaged losartan at a dose of 33.3 mg/kg/d for 10 weeks. Rats in the YSPDP-L and YSPDP-H groups were gavaged with YSPDP of 1.5 g/kg/d and 3 g/kg/d, Rats in the Sham and the 5/6 SNx + Vehicle groups were given an equal volume of ddH2O in the same way. 10 weeks after treatment, the animals were sacrificed and the blood, urine, and renal samples were collected for further analysis.

### 2.5 Biochemical parameters detection

Rat blood samples were allowed to clot at room temperature for 2 h. Subsequently, both blood and urine samples underwent centrifugation at 3,000 rpm for 10 min to collect the supernatant. The serum and urine samples were thereafter stored at −80°C until further analysis. Serum levels of albumin, alanine aminotransferase (ALT), aspartate aminotransferase (AST), Serum creatinine (Scr), blood urea nitrogen (BUN), and urine protein/creatinine were detected by Automatic Biochemistry Analyzer (Technicon, RA-1640, United States).

### 2.6 Histology and immunohistochemistry

Kidney tissues underwent fixation in 4% paraformaldehyde, followed by embedding in paraffin and sectioned at 4 μm thickness. Kidney sections were stained with Periodic acid-Schiff (PAS) and Masson’s trichrome as per the manufacturer’s instructions. Immunohistochemical staining involved overnight incubation at 4°C with primary antibodies, including anti-FN (F3648, Sigma-Aldrich, United States) and anti-Col-III (22734-1-AP, Proteintech). Subsequently, the sections were treated with biotinylated secondary antibodies for 1 h at 37°C, following standard protocols. Post-staining with 3,3′-Diaminobenzidine and counterstaining with hematoxylin, the density of positively stained areas was determined using Image-Pro Plus software.

### 2.7 Western blot analysis

Total tissue proteins were extracted using RIPA buffer (Beyotime Biotechnology, Shanghai, China). Proteins were separated using SDS-PAGE and transferred to PVDF membranes (Merck Millipore, MA, United States). The membranes were blocked with 5% BSA for 1 h, incubated with primary antibody overnight at 4°C, then incubated with secondary antibodies for 1 h, finally detected using enhanced chemiluminescence solution. The western blot images were analyzed by ImageJ software (National Institutes of Health, Bethesda, MD, United States). The following primary antibodies were used in this study: anti-FN (F3648, Sigma-Aldrich), anti-COl-I (14695*-*1-AP, Proteintech), anti-α-SMA (14395-1-AP, Proteintech), anti-mTOR (2983T, Cell Signaling Technology), anti-p-mTOR (5536T, Cell Signaling Technology), anti-p-PI3K (4228T, Cell Signaling Technology), anti-PI3K(20584-1-AP, Proteintech), anti-p-AKT (66,444-1- Ig, Proteintech), anti-AKT (9272T, Cell Signaling Technology), and anti-β-actin (20536-1-AP, Proteintech).

### 2.8 RNA sequencing analysis

Kidney tissue from the Sham, 5/6 SNx + Vehicle, and 5/6 SNx + YSPDP-H groups was used for transcriptomics sequencing. Transcriptomics sequencing was performed by Metware Biotechnology Co., Ltd. (Wuhan, China). We utilized DESeq2 to identify differentially expressed genes, applying the criteria of |log2(FoldChange)| > 1 and padj<0.05. For differential gene enrichment analysis, GOSeq and KOBAS were employed, focusing on corrected *p*-values < 0.05 for both GO and KEGG enrichment analyses.

### 2.9 Statistical analysis

All data are presented as Mean ± SEM. Statistical analyses were performed using GraphPad Prism 10.0 software (La Jolla, United States). The unpaired *t*-test was used to compare the differences between the two groups. Statistical significance was defined as *p* < 0.05.

## 3 Results

### 3.1 Characterization of chemical components in YSPDP

**FIGURE 1:**
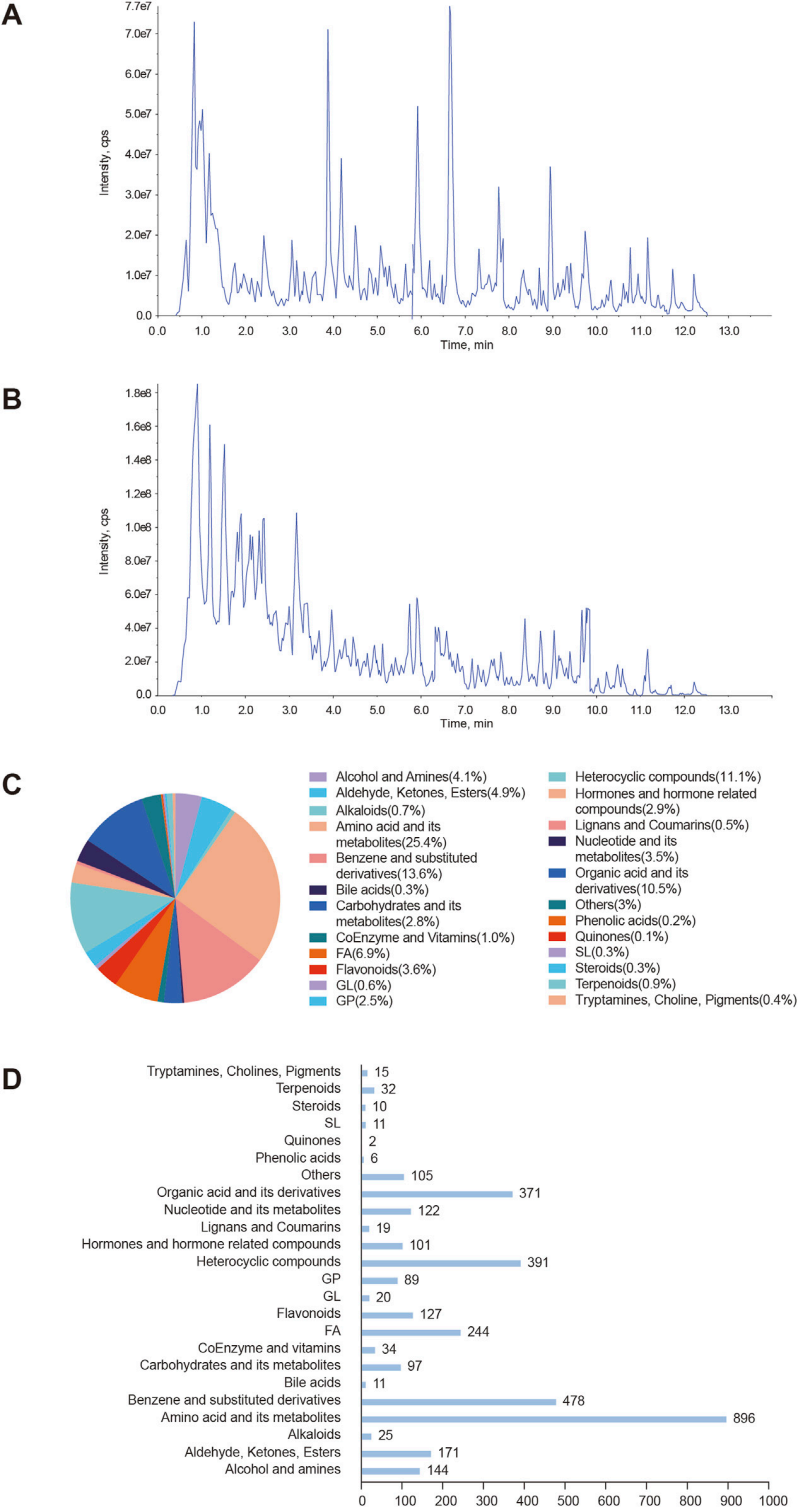
Characterization of chemical components in YSPDP **(A)**. The total ion current chromatogram in negative ion mode of YSPDP **(B)**. The total ion current chromatogram in positive ion mode of YSPDP **(C)**. The proportion of the twenty-four classes of chemical components in YSPDP **(D)**. The numbers of each class of chemical components in YSPDP.

Metabolomics was used to identify the chemical components of YSPDP. The total ion flow profiles in negative and positive ion mode for identifying compounds in the alcohol extract of YSPDP were shown in Figures 1A, B. Twenty-four classes of chemicals were identified. Among them, amino acid and its metabolites were the most abundant chemical class in YSPDP, which contained 896 chemicals (Figures 1C, D). Further, we listed the top abundant 20 chemicals in YSPDP, which were shown in Table 1.

**TABLE 1:** The top 20 abundant chemicals in YSPDP revealed by metabolomic.

Name	Formula	MW	Class
L-Proline	C5H9NO2	115.063	Amino acid and its metabolites
1-Aminocyclobutanecarboxylic acid	C5H9NO2	115.063329	Amino acid and its metabolites
2-Hydroxyadenosine	C10H13N5O5	283.0916,685	Nucleotide and its metabolites
Rhein	C15H8O6	284.0320,881	Benzene and substituted derivatives
5,7-Dimethoxyflavanone	C17H16O4	284.1,048,591	Aldehyde, Ketones,Esters
3-Hydroxycinnamic acid	C9H8O3	164.047345	Organic acid and its derivatives
Lunarine	C25H31N3O4	437.2,314,566	Heterocyclic compounds
Liquiritin	C21H22O9	418.1,263,824	Flavonoids
PC(16:0/2:0)	C26H52NO8P	537.343,056	PC(16:0/2:0)
Adrenosterone	C19H24O3	300.172,545	Hormones and hormone related compounds
Metribuzin	C8H14N4OS	214.0888,318	Heterocyclic compounds
Deethylatrazine	C6H10ClN5	187.0624,731	Heterocyclic compounds
4-Chloro-L-phenylalanine	C9H10ClNO2	199.0400,063	Amino acid and its metabolites
L-Phenylalanine	C9H11NO2	165.079	Amino acid and its metabolites
Chrysin	C15H10O4	254.0579,089	Flavonoids
Gefitinib	C22H24ClFN4O3	446.1,520,967	Heterocyclic compounds
(9R,13R)-10,11-dihydro-12-oxo-15-phytoenoic acid	C18H30O3	294.2,194,949	FA
Eicosanoyl-EA	C22H45NO2	355.345,029	Organic acid and its derivatives
3-Hydroxypyruvic acid	C3H4O4	104.01096	Organic acid and its derivatives
11,12,14-Trihydroxy-7-methoxy-8,11,13-abietatrien-20,6-olide	C21H28O6	376.1,885,888	Benzene and substituted derivatives

### 3.2 Network pharmacology prediction of YSPDP for CKD

**FIGURE 2:**
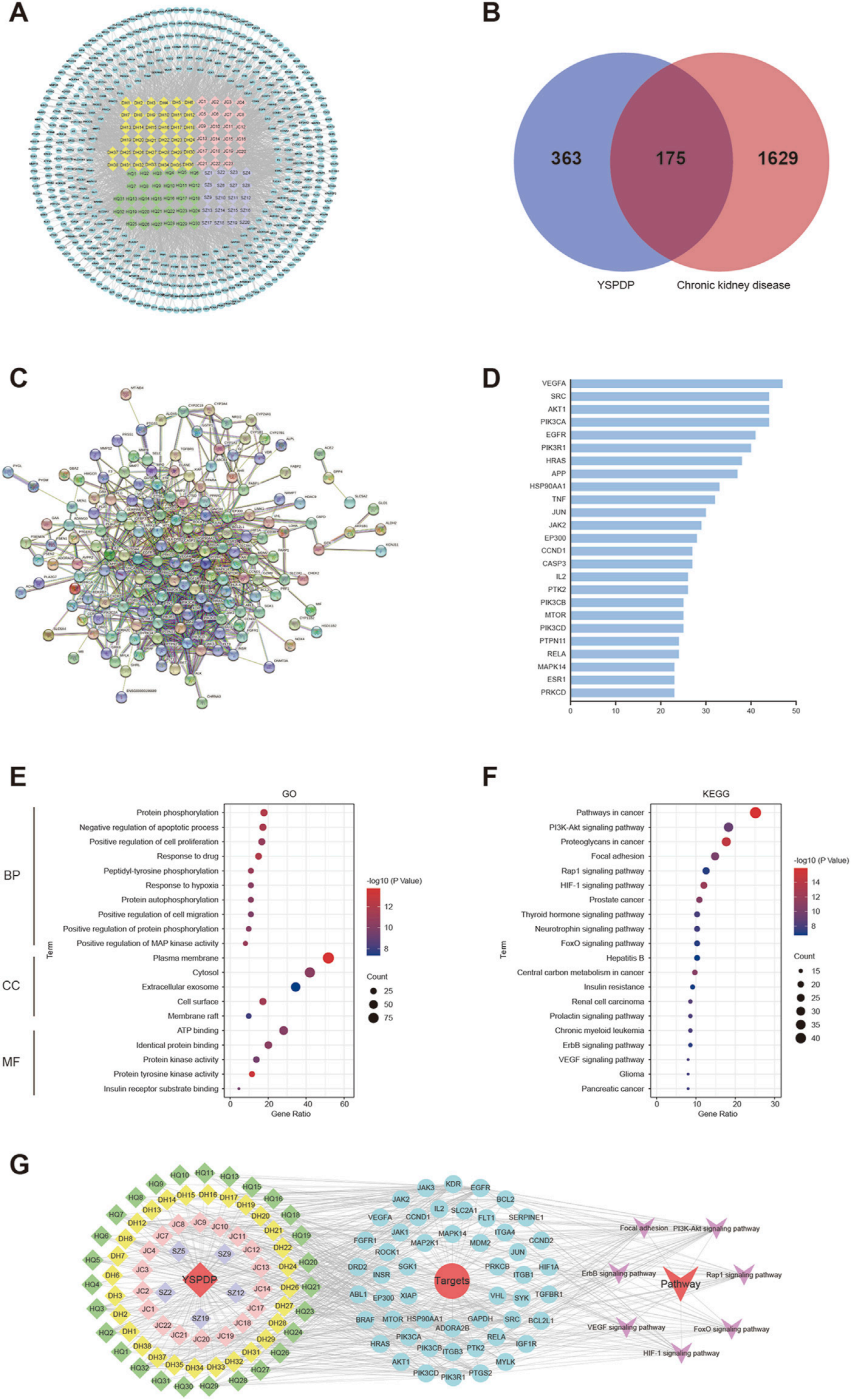
Network pharmacology analysis of YSPDP treatment of CKD **(A)**. The component-target network of YSPDP (The rhombuses represent the active components of Rheum officinale (yellow), Astragalus membranaceus (green), Bombyx batryticatus (pink) and Hirudo (purple); the blue circles represent the targets of the active components of YSPDP) **(B)**. Venn diagram of intersected targets of YSPDP and CKD **(C)**. The protein-protein interaction network of drug-disease intersected targets **(D)**. The top 25 targets in action frequency **(E)**. GO enrichment analysis. The top 10 significantly enriched terms of BP and the top 5 significantly enriched terms of CC and MF **(F)**. Top 20 enriched KEGG signaling pathways **(G)**. The “compounds-targets-pathways” network of YSPDP treatment of CKD. BP, biological process; CC, cell component; MF, molecular function.

To explore the therapeutic mechanism of YSPDP in CKD, network pharmacology was employed to predict its potential targets. The primary herbal ingredients in YSPDP include Rheum officinale, Astragalus membranaceus, Bombyx batryticatus, and Hirudo. Based on a criterion requiring an OB according to Veber’s filter, a DL following Lipinski rule of 5, and a high GI absorption, 127 compounds were determined from YSPDP. Among them, 45 compounds were in Rheum officinale, 30 compounds were in Astragalus membranaceus, 18 compounds were in Bombyx batryticatus, and 25 compounds were in Hirudo. 538 potential targets of these chemicals were obtained using the Swiss Target Prediction website (Figure 2A). The 175 potential targets of YSPDP exhibited significant alterations in the kidney of CKD (Figure 2B). 175 intersection targets were input into STING to construct PPI networks (Figure 2C) depicting YSPDP’s interference with CKD-related target genes. Based on the degree value of nodes, we further selected the top ten targets as the hub genes, including VEGFA, SRC, PIK3CA, AKT1, EGFR, PIK3R1, HRAS, APP, HSP90AA1, and TNF. The top 25 targets in action frequency are shown in Figure 2D. The DAVID tool website were used to analyze GO function annotation and KEGG pathway enrichment. GO enrichment analysis indicated that the biological processes of YSPDP on CKD were related to the negative regulation of the apoptotic process, positive regulation of cell proliferation, response to hypoxia, positive regulation of MAP kinase activity, and positive regulation of cell migration (Figure 2E). KEGG enrichment analysis revealed that the pathways of YSPDP on CKD were predominantly involved in PI3K-AKT, Rap1, HIF-1, FoxO, ErbB and VEGF signaling pathway and focal adhesion (Figure 2F). Finallly, based on these signaling pathway, the network of “compounds-targets-pathways” was constructed to reveal the therapeutic targets and pharmacological mechanisms of YSPDP against CKD (Figure 2G).

### 3.3 YSPDP improved biochemical parameters in 5/6 SNx rats

**FIGURE 3:**
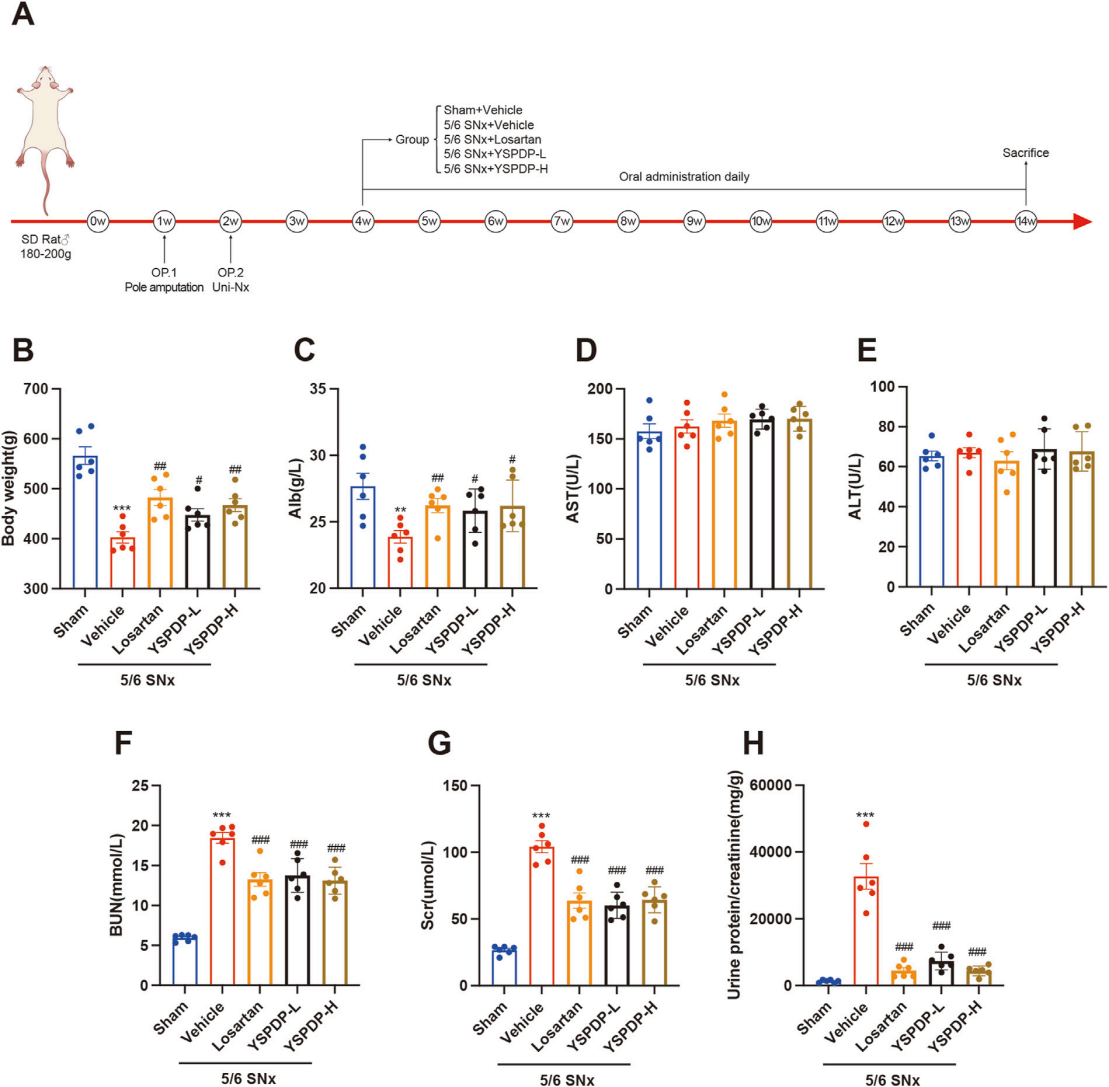
YSPDP improved biochemical parameters in 5/6 SNx rats **(A)**. Schematic representation of experimental animal research design. B-H. The body weight **(B)**, Alb **(C)**, AST **(D)**, ALT **(E)**, BUN **(F)**, Scr **(G)**, and Urine protein/creatinine **(H)** levels at the end of week 14 in rats from Sham, 5/6 SNx, Losartan (33.3 mg/kg/d) treated, YSPDP-low dose (1.5 g/kg/d) and YSPDP-high dose (3 g/kg/d) treated groups. Values are presented as the means ± SEM (n = 6). The symbols * and # represent statistical comparisons with the Sham and 5/6 SNx + Vehicle groups, respectively. * /#P < 0.05; **/## P < 0.01; *** /### P < 0.001.

To evaluate the effect of YSPDP on CKD, we created a murine model of 5/6 SNx and then treated rats with YSPDP for 10 weeks, starting at 2 weeks after 5/6 SNx. The flowchart of the study is illustrated in Figure 3A. 5/6 SNx caused a decrease in body weight and albumin (Alb), while losartan and YSPDP treatment led to restoration of body weight and Alb (Figures 3B, C). There were no significant differences in the aspartate aminotransferase (AST) and alanine aminotransferase (ALT) of all rats (Figures 3D, E), indicating YSPDP has no side effects on liver function. As shown in Figures 3F–H, serum creatinine (Scr), blood urea nitrogen (BUN), and urine protein/creatinine levels were higher in the 5/6 SNx group than in the Sham group. Both Losartan and YSPDP significantly ameliorated Scr, BUN, and urine protein/creatinine. The efficacy of YSPDP in ameliorating the progression of CKD was comparable to that of losartan. Furthermore, there were no statistically significant differences observed between the YSPDP-high dose (YSPDP-H) and YDSPDP-low dose (YSPDP-L) treated groups. In conclusion, YSPDP effectively improved the renal function of 5/6 SNx-induced CKD rats.

### 3.4 YSPDP improved the pathological damage in 5/6 SNx rats

**FIGURE 4:**
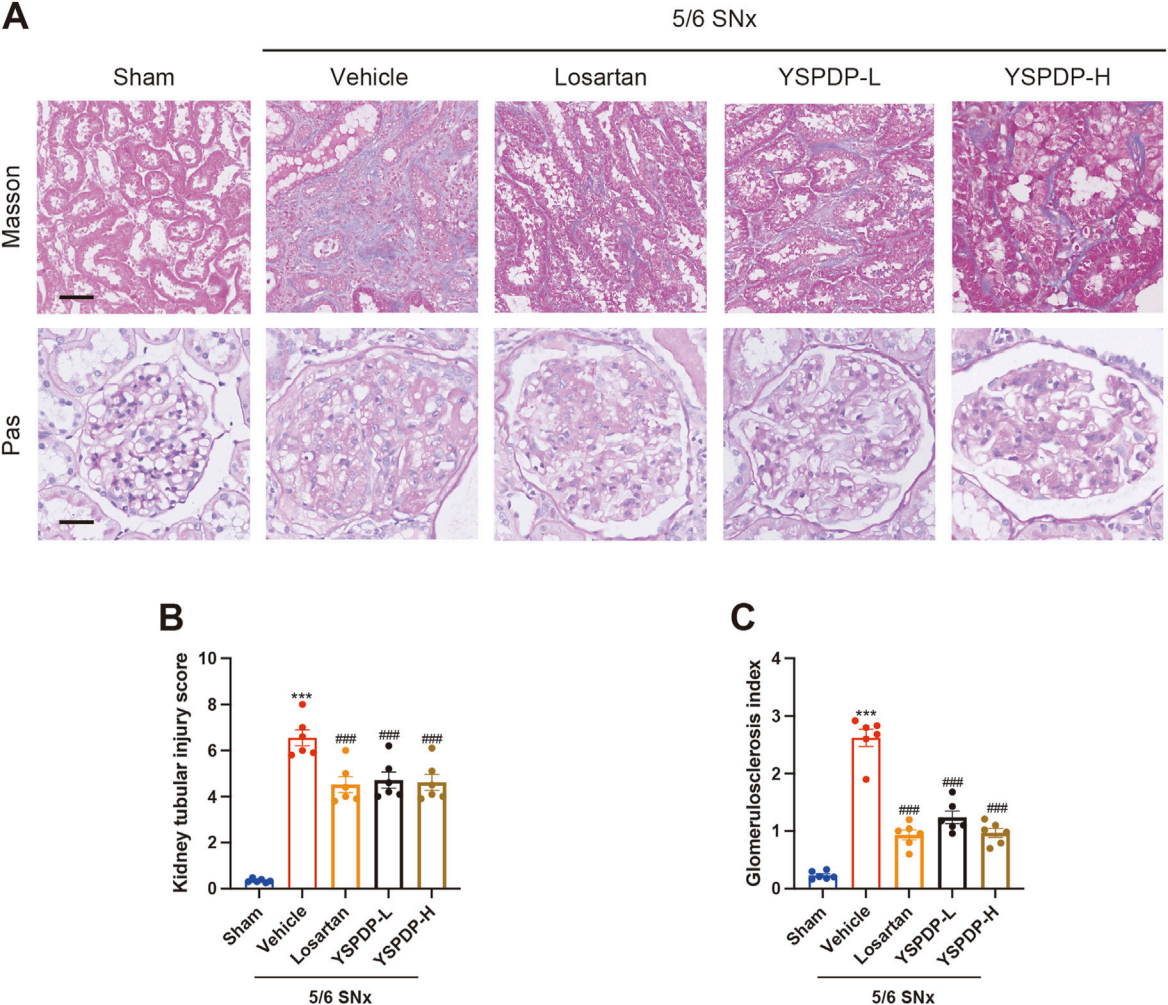
YSPDP improved the pathological damage in 5/6 SNx rats **(A)**. Photomicrographs illustrating Masson and Pas staining of kidney tissue. The top panel of Masson staining showed renal tubular injury in indicated groups. The bottom panel of PAS staining showed glomerular sclerosis in indicated groups. Scar bar = 50 μm **(B)**. Kidney tubular injury score based on Masson staining (n = 6) **(C)**. Glomerulosclerosis index based on PAS staining (n = 6). The symbols * and # represent statistical comparisons with the Sham and 5/6 SNx + Vehicle groups, respectively. * /#*P* < 0.05; **/##*P* < 0.01; *** /### *P* < 0.001.

To further evaluate the effect of YSPDP on renal function and structure, we examined renal pathology in these groups using Masson and PAS staining (Figure 4A). From the representative images of Masson staining, the 5/6 SNx group showed tubular degeneration, luminal dilation, and ECM deposition in the renal interstitium. The presence of glomerular sclerosis in the kidneys of the 5/6 SNx group was demonstrated through PAS staining. However, treatment with losartan, YSPDP-L, and YSPDP-H effectively mitigated tubular injury and glomerular sclerosis in 5/6 SNx rats (Figures 4B, C).

### 3.5 YSPDP hampers renal fibrosis in 5/6 SNx rats

**FIGURE 5:**
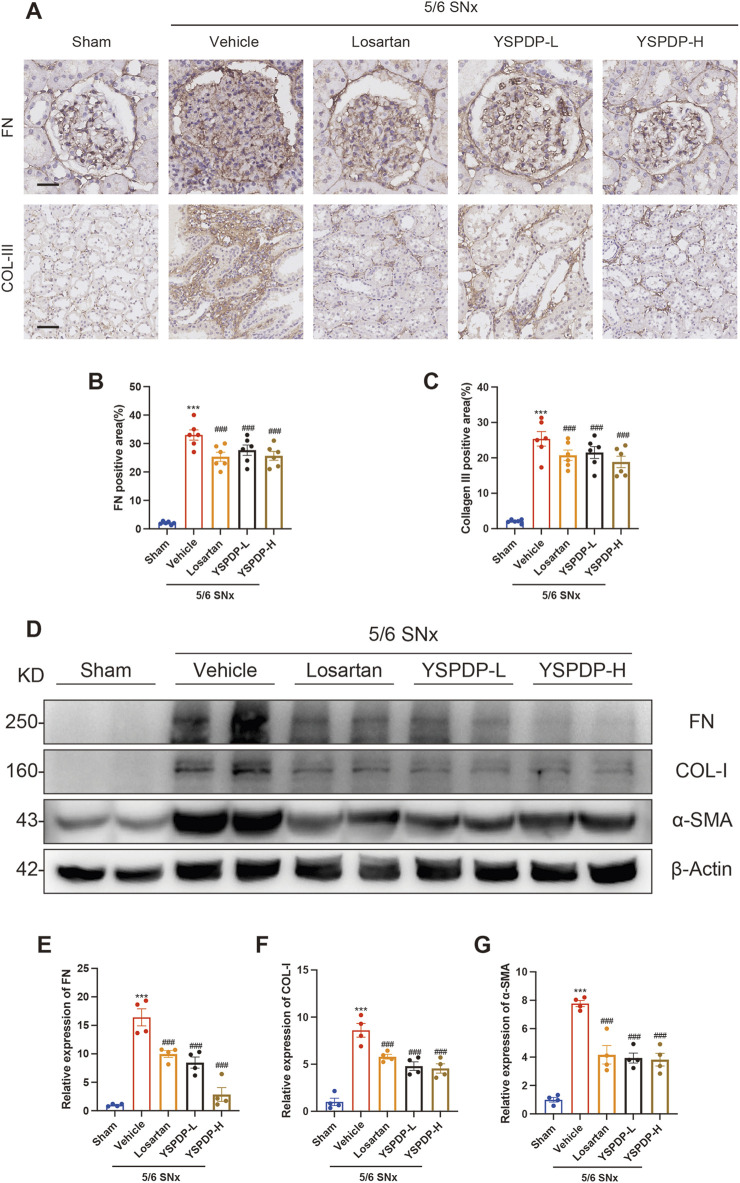
YSPDP hampers renal fibrosis in 5/6 SNx rats **(A)**. Immunohistochemical staining of FN and collagen III. Scale bar = 50 µm **(B, C)**. The percentage of FN **(B)** and collagen III **(C)** positive areas relative to the whole area was quantified (n = 6) **(D)**. Whole kidney tissue lysates were subjected to immunoblot analysis with specific antibodies against FN, collagen I, α-SMA, or β-Actin. Expression levels of FN **(E)**, and collagen I **(F)**, α-SMA **(G)** were quantified by densitometry analysis and then normalized with β-Actin (n = 4). The symbols * and # represent statistical comparisons with the Sham and 5/6 SNx + Vehicle groups, respectively. * /#*P* < 0.05; **/## *P* < 0.01; ***/### *P* < 0.001.

Immunohistochemical staining analysis showed that Fibronectin (FN) expression in the glomerulus in 5/6 SNx group was significantly increased, while losartan, YSPDP-L, and YSPDP-H treatment effectively decreased the glomerular deposition of FN in 5/6 SNx rats (Figure 5A). Immunohistochemical staining of Collagen III (COL-III) showed that renal tubulointerstitial COL-III expression in the 5/6 SNx group was significantly enhanced and residual renal tissue staining in the losartan, YSPDP-L, and YSPDP-H treated groups was reduced (Figure 5B). The quantitative analysis of the FN and COL-III staining intensity and area showed that the expression of FN and COL-III was decreased by losartan, YSPDP-L, and YSPDP-H treatment significantly (Figures 5C, D). Western blotting showed that FN, collagen I (COL-I) and α-SMA protein expression in the 5/6 SNx group was significantly increased and that their expression in the losartan, YSPDP-L, and YSPDP-H treated groups was decreased (Figures 5D–G).

### 3.6 Transcriptomics analysis results of 5/6 SNx rats treated with YSPDP

**FIGURE 6:**
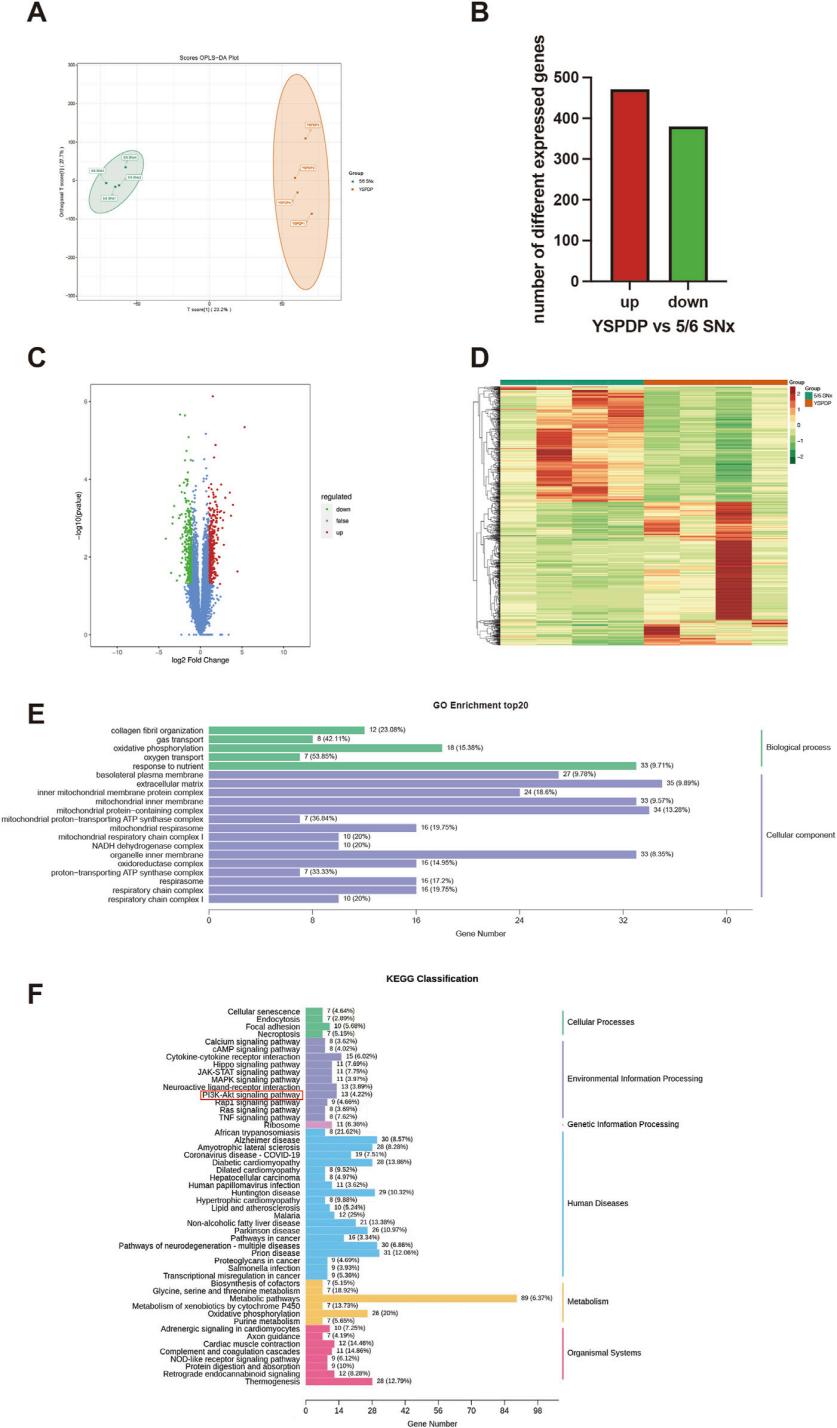
Transcriptomic analysis results of 5/6 SNx rats treated with YSPDP **(A)**. OPLS-DA score plot of transcriptomic analysis in YSPDP (5/6 SNx + YSPDP-H) and 5/6 SNx (5/6 SNx + Vihicle) group (n = 4) **(B)**. Identification of DEGs (q-value <0.05, |log_2_FC| ≥ 1) **(C)**. Volcano plots of DEGs; **(D)**. Heatmap of DEGs **(E)**. GO enrichment analysis. The top 5 significantly enriched terms of BP and the top 15 significantly enriched terms of CC **(F)**. Top 50 enriched KEGG signaling pathways.

To further analyze the underlying mechanism of YSPDP in CKD, the kidney tissues of 5/6 SNx rats treated with vehicle and YSPDP-H were performed with transcriptome sequencing. The OPLS-DA analyses showed the gene expression between the two groups was quite different from each other (Figure 6A). 851 differentially expressed genes (DEGs) were found in the YSPDP group, including 471 upregulated and 380 downregulated genes compared to the 5/6 SNx group (Figures 6B–D). The DEGs were subsequently analyzed using GO and KEGG databases. The enrichment analysis results revealed that YSPDP could regulate several molecular functions, such as collagen fibril organization, oxidative phosphorylation, and oxygen transport, and was involved in important biological processes, including extracellular matrix organization and inflammatory response (Figure 6E). KEGG pathway enrichment analysis further demonstrated that the DEGs were enriched in various inflammation- and apoptosis-related pathways including the PI3K-AKT signaling pathway (Figure 6F).

### 3.7 YSPDP inhibited the PI3K/AKT/mTOR signaling pathway of 5/6 SNx rats

**FIGURE 7:**
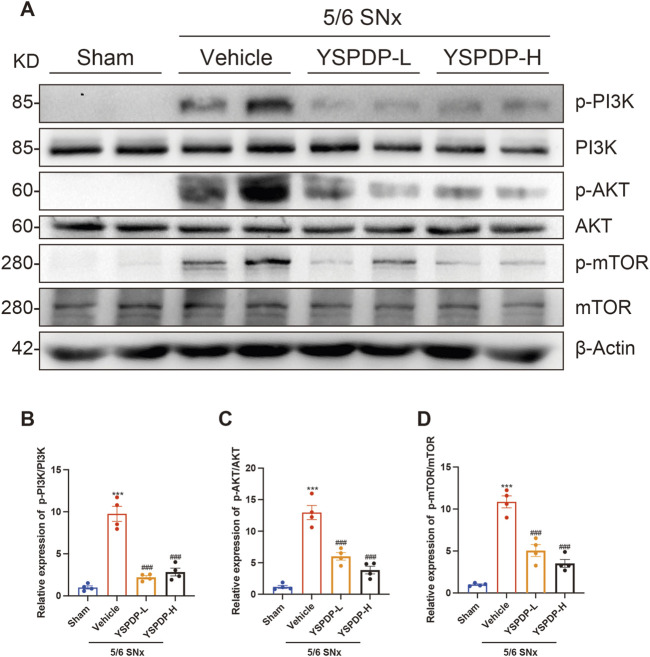
YSPDP inhibited the PI3K/AKT/mTOR signaling pathway of 5/6 SNx rats **(A)**. Whole kidney tissue lysates were subjected to immunoblot analysis with specific antibodies against p-PI3K, PI3K, p-AKT, AKT, p-mTOR, mTOR, or β-Actin. **(B-D)**. Relative protein levels of p-PI3K/PI3K **(B)**, p-AKT/AKT **(C)**, p-mTOR/ mTOR **(D)** (n = 4). The symbols * and # represent statistical comparisons with the Sham and 5/6 SNx + Vehicle groups, respectively. * /#*P* < 0.05; **/## *P* < 0.01; *** /### *P* < 0.001.

Based on the network pharmacology and Transcriptomics, we propose that the PI3K/AKT signaling pathway may serve as the primary mechanism by which YSPDP exerts its therapeutic effects in CKD. This pathway is crucial for regulating cell survival, proliferation, and apoptosis, all of which are critical processes in the progression of CKD. Moreover, activation of the PI3K/AKT pathway has been implicated in mediating renal inflammation and fibrosis, which are key features of CKD pathophysiology (Li et al., 2017; Zhang et al., 2021; Wang et al., 2024). To investigate this further, we examined the expression of key proteins in the PI3K/AKT pathway in 5/6 SNx rats. Compared to the sham group, the phosphorylation levels of PI3K, AKT, and mTOR were significantly increased following 5/6 SNx treatment. Importantly, the phosphorylation levels of PI3K, AKT, and mTOR induced by 5/6 SNx were significantly decreased by YSPDH treatment (Figures 7A– D). These results suggest that the therapeutic effects of YSPDP in CKD are closely associated with the modulation of the PI3K/AKT/mTOR pathway.

## 4 Discussion

CKD is a complex, multifactorial condition that can arise from various causes, including hypertension, diabetes, and primary glomerulonephritis (Zhang et al., 2012). Once CKD progresses to ESRD, the only available therapeutic option is renal replacement therapies, such as dialysis or kidney transplantation (Liu, 2013). These treatments are not only expensive but are also associated with a high burden of morbidity and mortality (Ng and Li, 2018). Therefore, the exploration of novel therapeutic options, especially those that can halt or reverse the progression of CKD and prevent the onset of ESRD, is critical for improving patient outcomes.

In recent years, a growing body of evidence has demonstrated the therapeutic potential of natural products in the management of CKD, particularly in combatting renal fibrosis, the hallmark of disease progression (Chen et al., 2018). Renal fibrosis is characterized by excessive deposition of extracellular matrix (ECM) components, leading to structural damage and functional impairment of the kidney. Research into traditional Chinese medicine (TCM) has shown that various herbs, herbal extracts, and TCM formulations can slow or even reverse renal fibrosis (Li et al., 2022).

In this study, we focused on YSPDP, a well-established TCM remedy that has been used clinically in Wuhan Union hospital for the treatment of CKD. YSPDP is composed of four key herbs: *Rheum officinale, Astragalus membranaceus, Bombyx batryticatus,* and *Hirudo*. These herbs are known for their anti-inflammatory, anti-fibrotic, and kidney-protective properties (Wang et al., 2012; Yang et al., 2021; Shen et al., 2023). Given its strong clinical efficacy, YSPDP has garnered interest as a potential therapeutic agent for CKD, prompting us to investigate the underlying mechanisms of its renal protective effects.

To elucidate the pharmacological basis of YSPDP’s efficacy, we identify some active components of this formulation including Nicotinamide, Rutin, Kaempferol, Astragaloside IV, Ursolic Acid, Emodin, Rhein, Quercetin, and Hederagenin. These compounds have been reported to exert anti-renal fibrosis effects through various mechanisms, such as autophagy regulation, antioxidant activity, anti-inflammation, and apoptosis modulation (Wang et al., 2016; Wang et al., 2023; Lu et al., 2018; Thakur et al., 2018; Wu et al., 2020; Kumakura et al., 2021; Guan et al., 2023; Jia et al., 2023; Li et al., 2023). The identification of these bioactive compounds provides compelling evidence supporting the renal protective function of YSPDP, as these compounds target key pathways involved in fibrosis and inflammation.

We utilized The 5/6 SNx model, which is widely acknowledged as the classical model that most closely resembles human CKD (Kim et al., 2021). Our findings demonstrated that treatment with YSPDP markedly hindered the progression of CKD, as evidenced by restoration of body weight and serum Alb levels, improved renal function, and diminished renal injury in 5/6 SNx rats. Specifically, YSPDP decreased proximal tubule atrophy, limited inflammatory cell infiltration, reduced collagen accumulation, and inhibited fibrous tissue proliferation. Renal fibrosis is the common outcome of most progressive CKD, irrespective of the underlying causes, and closely correlates with the decline in renal function (Liu, 2011), these findings suggest that YSPDP has broad reno-protective effects.

To explore the molecular mechanisms underlying these protective effects, we examined key fibrotic markers, including FN, COL-I, COL-III, and α-SMA, using immunohistochemistry and Western blot analysis. YSPDP treatment significantly reduced the expression of these fibrotic markers, further supporting its role in attenuating renal fibrosis.

Network pharmacology was employed to explore the potential active ingredients and molecular targets of YSPDP. By intersecting the potential targets of YSPDP with CKD-associated targets, we constructed a protein interaction network. The top 10 targets identified included key signaling molecules such as VEGFA, SRC, PIK3CA, AKT1, EGFR, PIK3R1, HRAS, APP, HSP90AA1, and TNF. GO functional analysis and KEGG enrichment analysis revealed that the PI3K/AKT signaling pathway is central to the mechanism of YSPDP’s anti-CKD effects. These results were further supported by transcriptomics data, which indicated that YSPDP modulates multiple cellular processes involved in renal fibrosis and inflammation.

The PI3K/AKT pathway plays a critical role in regulating a wide range of cellular functions, including cell proliferation, survival, differentiation, and metabolism (Yu and Cui, 2016). PI3K is an enzyme involved in neural signal transduction, activated by various receptors such as tyrosine kinase, G protein-coupled cytokine, and Ras-associated GDP enzyme receptors. This activation promotes processes like cell proliferation, survival, adhesion, differentiation, and cytoskeletal organization (Yu et al., 2022). AKT, a downstream target of PI3K, is crucial in regulating multiple cellular functions, including proliferation, apoptosis, glucose metabolism, cell migration, and transcription (Manning and Toker, 2017). The phosphorylation of the PI3K/AKT/mTOR signaling cascade modulates various downstream effector molecules, influencing several biological processes in kidney tissue, such as cell death, lipid metabolism, and epithelial-mesenchymal transition (EMT). These processes contribute directly to the advancement of renal fibrosis and CKD (Lee et al., 2007). In our research, we observed elevated phosphorylation levels of PI3K, AKT, and mTOR in the kidneys of CKD model rats, indicating activation of this signaling cascade. Importantly, YSPDP treatment significantly reduced the phosphorylation of these proteins, suggesting that YSPDP exerts its protective effects by inhibiting the PI3K/AKT/mTOR pathway.

In addition to the PI3K/AKT pathway, transcriptomics analysis also showed that YSPDP may influence cellular senescence. Research has demonstrated that the cellular senescence of renal tubular epithelial cells plays a key role in driving renal fibrosis. Slowing down this process is considered an effective approach to mitigating renal fibrosis and is a crucial strategy for delaying the progression of CKD (Li et al., 2021; Wang et al., 2021). YSPDP may prevent renal fibrosis by delaying cell senescence, which needs further verification.

Our study showed the efficacy of YSPDP in ameliorating the progression of CKD was comparable to that of losartan. Clinically, some patients exhibit resistance to renin-angiotensin system inhibitors or experience significant intolerance toward severe side effects associated with these medications, such as hyperkalemia, hypotension, and even renal failure (Laurent, 2017). However, in our clinical practice, YSPDP has demonstrated a lack of these adverse effects. In subsequent studies, we will focus on the adverse drug reactions and long-term prognosis of YSPDP to CKD.

Although this study provides important insights into the mechanism by which YSPDP alleviates renal fibrosis, several limitations warrant further investigation. First, CKD can result from a wide range of etiologies, and it is essential to test YSPDP’s efficacy in other CKD models, such as diabetic nephropathy, to determine its broader applicability. Second, although we identified multiple bioactive compounds in YSPDP, future studies should focus on elucidating the specific roles of these individual compounds in mediating the observed effects. Finally, genetic knockout models could provide more definitive evidence regarding the molecular pathways through which YSPDP exerts its reno-protective effects. Addressing these limitations in future research will help to solidify YSPDP’s potential as a novel therapeutic option for CKD.

In conclusion, our study highlighted the protective effects of YSPDP in a 5/6 SNx model using SD rats. By integrated network pharmacology, transcriptomic analysis, and pharmacological assessment, we predicted and confirmed that the therapeutic mechanism of YSPDP in addressing 5/6 SNx-induced renal fibrosis may involve inhibition of the PI3K/AKT/mTOR signaling pathway. These findings suggest that YSPDP could be a potential therapeutic candidate for managing renal fibrosis.

## Data Availability

The transcriptomic data presented in the study are deposited in the NCBI database, accession number PRJNA1172152 (https://www.ncbi.nlm.nih.gov/sra/PRJNA1172152).
