# Supplementary material for: Yishen paidu pills attenuates 5/6 nephrectomy induced kidney disease via inhibiting the PI3K/AKT/mTOR signaling pathway
Source: Front Pharmacol. 2024 Nov 28;15:1510098. doi: 10.3389/fphar.2024.1510098 (PMC11634598; doi:10.3389/fphar.2024.1510098)
Supplement: Supplementary file 1 [file DataSheet1.DOCX]

**Supplementary File 1. Metabolomics profiling**

1.Sample preparation and extraction

  The sample stored at -80 °C refrigerator was thawed on ice. The sample was grinded with liquid nitrogen. A 400 μL solution (Methanol : Water = 7:3, V/V) containing internal standard was added into 20 mg grinded sample, and shaked at 1500 rpm for 5 min. After placing on ice for 15 min. the sample was centrifuged at 12000 rpm for 10 min (4 °C). A 300 μL of supernatant was collected and placed in -20 °C for 30 min. The sample was then centrifuged at 12000 rpm for 3 min (4 °C). A 200 μL aliquots of supernatant were transferred for LC-MS analysis.

2. T3 UPLC Conditions

  T3 UPLC Conditions : The sample extracts were analyzed using an LC-ESI-MS/MS system (UPLC, ExionLC AD，<https://sciex.com.cn/>; MS, QTRAP® System, <https://sciex.com/>). The analytical conditions were as follows, UPLC: column, Waters ACQUITY UPLC HSS T3 C18 (1.8μm, 2.1 mm*100 mm); column temperature, 40°C; flow rate, 0.4 mL/min; injection volume, 2μL or 5μL; solvent system, water (0.1% formic acid): acetonitrile (0.1% formic acid); gradient program, 95:5 V/V at 0 min, 10:90 V/V at 10.0 min, 10:90 V/V at 11.0 min, 95:5 V/V at 11.1 min, 95:5 V/V at 14.0 min.

3. QTOF-MS/MS

  The Triple TOF mass spectrometer was used for its ability to acquire MS/MS spectra on an information-dependent basis (IDA) during an LC/MS experiment. In this mode, the acquisition software (TripleTOF 6600, AB SCIEX) continuously evaluates the full scan survey MS data as it collects and triggers the acquisition of MS/MS spectra depending on preselected criteria. In each cycle, 12 precursor ions whose intensity greater than 100 were chosen for fragmentation at collision energy (CE) of 30 V (12 MS/MS events with product ion accumulation time of 50 msec each). ESI source conditions were set as following: Ion source gas 1 as 50 Psi, Ion source gas 2 as 50 Psi, Curtain gas as 25 Psi, source temperature 500°C, Ion Spray Voltage Floating (ISVF) 5500 V or -4500 V in positive or negative modes, respectively.

4. ESI-Q TRAP-MS/MS

  LIT and triple quadrupole (QQQ) scans were acquired on a triple quadrupole-linear ion trap mass spectrometer (QTRAP), QTRAP® LC-MS/MS System, equipped with an ESI Turbo Ion-Spray interface, operating in positive and negative ion mode and controlled by Analyst 1.6.3 software (Sciex). The ESI source operation parameters were as follows: source temperature 500°C; ion spray voltage (IS) 5500 V (positive), -4500 V (negative); ion source gas I (GSI), gas II (GSII), curtain gas (CUR) were set at 50, 50, and 25.0 psi, respectively; the collision gas (CAD) was high. Instrument tuning and mass calibration were performed with 10 and 100 μmol/L polypropylene glycol solutions in QQQ and LIT modes, respectively. A specific set of MRM transitions were monitored for each period according to the metabolites eluted within this period.
